# Ilamycin C induces apoptosis and inhibits migration and invasion in triple-negative breast cancer by suppressing IL-6/STAT3 pathway

**DOI:** 10.1186/s13045-019-0744-3

**Published:** 2019-06-11

**Authors:** Qing Xie, Zhijie Yang, Xuanmei Huang, Zikang Zhang, Jiangbin Li, Jianhua Ju, Hua Zhang, Junying Ma

**Affiliations:** 1 Department of Clinical Biochemistry, Institute of Clinical Laboratory Medicine, Guangdong Provincial Key Laboratory of Medical Molecular Diagnostics, Guangdong Medical University, Dongguan, 523808 China; 2 CAS Key Laboratory of Tropical Marine Bio-Resources and Ecology, Guangdong Key Laboratory of Marine Materia Medica, RNAM Center for Marine Microbiology, South China Sea Institute of Oceanology, Chinese Academy of Sciences, Guangzhou, 510301 China

**Keywords:** Ilamycin C, Triple-negative breast cancer, Apoptosis, Invasion, Migration, IL-6, STAT3

## Abstract

**Background:**

Triple-negative breast cancer (TNBC) is the most aggressive subtype of breast cancer with poor prognosis, and its treatment remains a challenge due to few targeted medicines and high risk of relapse, metastasis, and drug resistance. Thus, more effective drugs and new regimens for the therapy of TNBC are urgently needed. Ilamycins are a kind of cyclic peptides and produced by *Streptomyces atratus* and *Streptomyces islandicus* with effective anti-tuberculosis activity. Ilamycin C is a novel compound isolated from the deep South China Sea-derived *Streptomyces atratus* SCSIO ZH16 and exhibited a strong cytotoxic activity against several cancers including breast cancer cell line MCF7. However, the cytotoxic activity of Ilamycin C to TNBC cells and a detailed antitumor mechanism have not been reported.

**Methods:**

CCK-8 assays were used to examine cell viability and cytotoxic activity of Ilamycin C to TNBC, non-TNBC MCF7, and nonmalignant MCF10A cells. EdU assays and flow cytometry were performed to assess cell proliferation and cell apoptosis. Transwell migration and Matrigel invasion assays were utilized to assess the migratory and invading capacity of TNBC cells following the treatment of Ilamycin C. The expressions of proteins were detected by western blot.

**Results:**

In this study, we found that Ilamycin C has more preferential cytotoxicity in TNBC cells than non-TNBC MCF7 and nonmalignant MCF10A cells. Notably, our studies revealed the mechanism that Ilamycin C can induce Bax/Bcl-2-related caspase-dependent apoptosis and inhibit migration and invasion through MMP2/MMP9/vimentin/fascin in TNBC by suppressing IL-6-induced STAT3 phosphorylation.

**Conclusions:**

This study provides the first evidence that Ilamycin C has significant implications for the potential as a novel IL-6/STAT3 inhibitor for TNBC treatment in the future.

**Electronic supplementary material:**

The online version of this article (10.1186/s13045-019-0744-3) contains supplementary material, which is available to authorized users.

## Background

Triple-negative breast cancer (TNBC) is characterized by lack of progesterone receptor (PR), estrogen receptor (ER), and human epidermal growth factor receptor 2 (HER2), accounting for about 15–20% of all breast cancer [1]. Clinically, TNBC is more aggressive and less sensitive to typical therapies and ultimately has a higher rate of relapse and metastasis and poorer prognosis compared with other subtypes of breast cancer [2, 3]. Chemotherapy is the main treatment of TNBC, but chemotherapy resistance has become an inevitable problem [4]. Lack of well-defined molecular targets makes it a challenge to treat and improve the 5-year survival rate of patients with TNBC [5, 6]. Therefore, new regimens including drug development based on molecular targets or chimeric antigen receptor (CAR)-engineered T cell approach for the treatment of TNBC are urgently needed [7].

Signal transducer and activator of transcription-3 (STAT3) is continually activated in many human cancers [8]. It can be directly or indirectly activated by many elements, such as growth factors (PDGFR, EGFR, and HER2), cytokines (IFN-α, IL-6), and non-receptor tyrosine kinases (Src and Janus kinase family proteins) [9–11]. Among the Janus kinase (JAK) family, JAK2 can be activated by IL-6 and further recruits and phosphorylates STAT3, thus functioning as an intermediary between IL-6 and STAT3 [10]. Studies showed that activity of STAT3 is closely relevant to cancer progression including proliferation, apoptosis, and metastasis [12–16]. It has also been found that the abnormal activity of IL-6/STAT3 relates to poor prognosis and a low survival rate in TNBC; thus, effective STAT3 inhibitors have become promising candidate drugs for treatment of it [17]. Currently, marine-derived natural products have attracted great interest for their novel structure, diverse bioactivities, and new function mechanisms; therefore, it has become a treasure of leading compounds for the development of new drugs [18, 19]. The fact that more antitumor drugs approved by the FDA and many antitumor compounds entering preclinical and clinical research are derived from marine organisms has highlighted that natural products from marine organisms have provided a constant source for new drug discovery against cancers [20].

Ilamycins are a kind of cyclic peptides and produced by *Streptomyces atratus* and *Streptomyces islandicus* with an effective anti-tuberculosis activity [21]*.* Our previous study found that Ilamycin C (Additional file 1: Figure S1), a novel compound isolated from the deep South China Sea-derived *Streptomyces atratus* SCSIO ZH16, exhibited a strong cytotoxic activity against several cancers including non-TNBC cell line MCF7 [21]. However, the cytotoxic activity to TNBC cells and detailed antitumor mechanism are still unknown. In this work, the cytotoxicity and function of Ilamycin C in TNBC cells were investigated and its antitumor mechanism was further explored.

## Methods

### Compounds

The structural elucidation, biosynthesis, and purification method of Ilamycin C were described in our previous study [21]. The purity of Ilamycin C is 97.8% analyzed by HPLC (high-performance liquid chromatography) analysis, and it was dissolved in dimethyl sulfoxide (DMSO) (Sigma). Doxorubicin and cisplatin were purchased from Sigma.

### Cell culture

MDA-MB-231, BT-549, MCF7, and MCF10A cell lines were obtained from American Type Culture Collection (ATCC). All these cells were cultured according to ATCC recommendations.

### Cell infection with lentivirus

The lentivirus vector was purchased from GenePharma. All vectors were verified by DNA sequencing. The lentivirus-STAT3 (LV-STAT3) or lentivirus-negative control (LV-NC) was used to infect MDA-MB-231 and BT-549. After 72 h, cells were selected using 0.6 μg/ml puromycin-resistant culture (Sigma) for a week. Cells were collected, and the STAT3 expression was analyzed by quantitative polymerase chain reaction (qRT-PCR).

### Cell transfection with RNA interference

For STAT3 RNA interference (RNAi), siRNA duplexes (5′-CCAACGACCUGCAGCAAUA-3′) against STAT3 (si-STAT3) and control duplex (5′-CCUACGCCACCAAUUUCGU-3′) were purchased from GenePharma and transfected into the MDA-MB-231 and BT-549 using the Lipofectamine 3000 (Invitrogen) according to the manufacturer’s guidelines.

### Cell viability and proliferation assays

Cell viability was tested by Cell Counting Kit-8 (CCK-8, DojinDo). Cells were seeded at 3000 cells per well in 96-well plates in triplicate and cultured for 24 h; Ilamycin C was added for 48 h. All control groups contained 0.1% DMSO. Then, 10 μL CCK-8 was added to every well, and plates were incubated at 37 °C for 2–3 h. The absorbance was detected at 450 nm in a Spectra Max 190 Enzyme standard instrument (Molecular Devices). Cell proliferation was measured with Click-iT®EdU Flow Cytometry Assay Kits (Invitrogen).

### Apoptotic assays

2.0 × 10^5^ cells were seeded per well in six-well plates for 24 h, then treated with different concentrations of Ilamycin C for 12 or 24 h. After this, cells were stained with Annexin V-FITC and PI or Annexin V-APC and 7-AAD at room temperature for 15 min and then analyzed by flow cytometry (Becton Dickinson Company).

### Transwell assays

After treatment with Ilamycin C for 24 h, cells (2.0 × 10^5^ per well) were resuspended with serum-free medium and seeded on the top side of the filters with 8-μm pore size (Millipore) and the low side was added with 10% FBS medium. Only invasion assays need to be precoated with Matrigel. Transwell migration and invasion assays were performed according to the manufacturer’s instructions. The images were taken by an inverted microscope (Olympus).

### Western blot analysis

Cells were treated with different concentrations of Ilamycin C for 24 h. Total proteins were obtained after the disposition of cells with RIPA lysis buffer together with protease inhibitors, phosphatase inhibitors, and PMSF (Beyotime, China). BCA assays were used for protein quantification. Proteins were separated by electrophoresis on a 12% SDS-polyacrylamide gel, electroblotted onto a PVDF membrane (BioRad Laboratories), and incubated with anti-Bcl-xL, anti-caspase-3, anti-caspase-7, anti-caspase-9, anti-STAT3, anti-vimentin, anti-p-STAT3, anti-β-actin, anti-Histone H3 (Cell Signaling Technology), anti-Bcl-2, anti-Bax, anti-Fascin (Abcam), anti-PARP1, anti-MMP2, and anti-MMP9 (Proteintech). Immunoreactivity was determined by using a Chemi DOC™ XRS+ system (BioRad Laboratories).

### Statistical analysis

All data were analyzed by GraphPad Prism 5.0 software. Results are shown as mean ± SD from three independent experiments. ANOVA and *t* test were appropriately used. *p* < 0.05 (*) was considered significant.

## Results

### Ilamycin C shows a preferential cytotoxic activity against TNBC cells

**Fig. 1:**
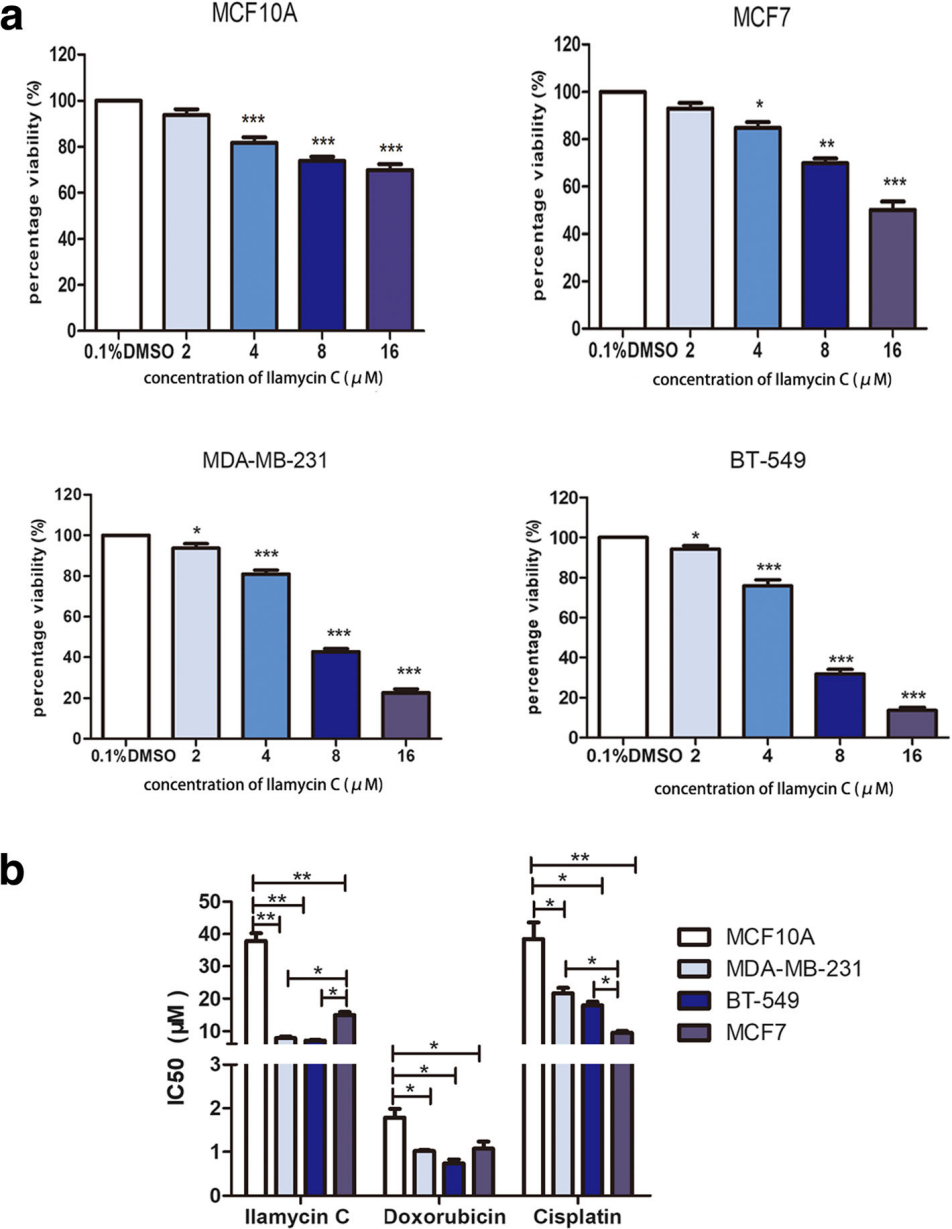
The cytotoxic activity of Ilamycin C in breast cancer and nonmalignant cell lines. **a** The percentages of cell viability with the treatment of increasing concentrations of Ilamycin C for 48 h. **b** The IC_50_ values of breast cancer and nonmalignant cell lines with the treatment of Ilamycin C, doxorubicin, and cisplatin for 48 h. Experiments were performed in triplicates. ***p < 0.001, **p < 0.01, *p < 0.05

To investigate whether Ilamycin C has a better cytotoxic effect on TNBC, the cytotoxic activity of Ilamycin C was examined using a Cell Counting Kit-8 (CCK-8) assay in two TNBC cell lines (MDA-MB-231 and BT-549), non-TNBC cell line (MCF7), and normal breast epithelial cell line (MCF10A). 0.1% DMSO was used as vehicle control. As showed in Fig. 1a, the percentages of cell viability in both MDA-MB-231 and BT-549 were sharply reduced compared with MCF7 and MCF10A cells after the treatment of Ilamycin C with increasing concentrations for 48 h, especially at 8 μM and 16 μM. Fifty percent inhibitory concentration (IC_50_) values were calculated to show the cytotoxic activity of Ilamycin C (Fig. 1b). The IC_50_ value of MCF7 was 15.93 μM, and the nonmalignant MCF10A cell line showed an IC_50_ value of 35.53 μM. However, the MDA-MB-231 and BT-549 cells exhibited similar mean IC_50_ values of 7.26 μM and 6.91 μM, respectively. Doxorubicin and cisplatin were used to compare the cytotoxic activity with Ilamycin C. The IC_50_ values showed that doxorubicin had strong cytotoxic activity to normal breast epithelial cell MCF10A and similar cytotoxic activity to both TNBC cells (MDA-MB-231 and BT-549) and non-TNBC cells (MCF7) (Fig. 1b). Cisplatin showed a better cytotoxic activity to MCF7 cells than TNBC cells (Fig. 1b). These results revealed that compared with doxorubicin and cisplatin, Ilamycin C has a better cytotoxic activity against TNBC than non-TNBC MCF7 and nonmalignant MCF10A cells.

### Ilamycin C suppresses proliferation in TNBC cells

**Fig. 2:**
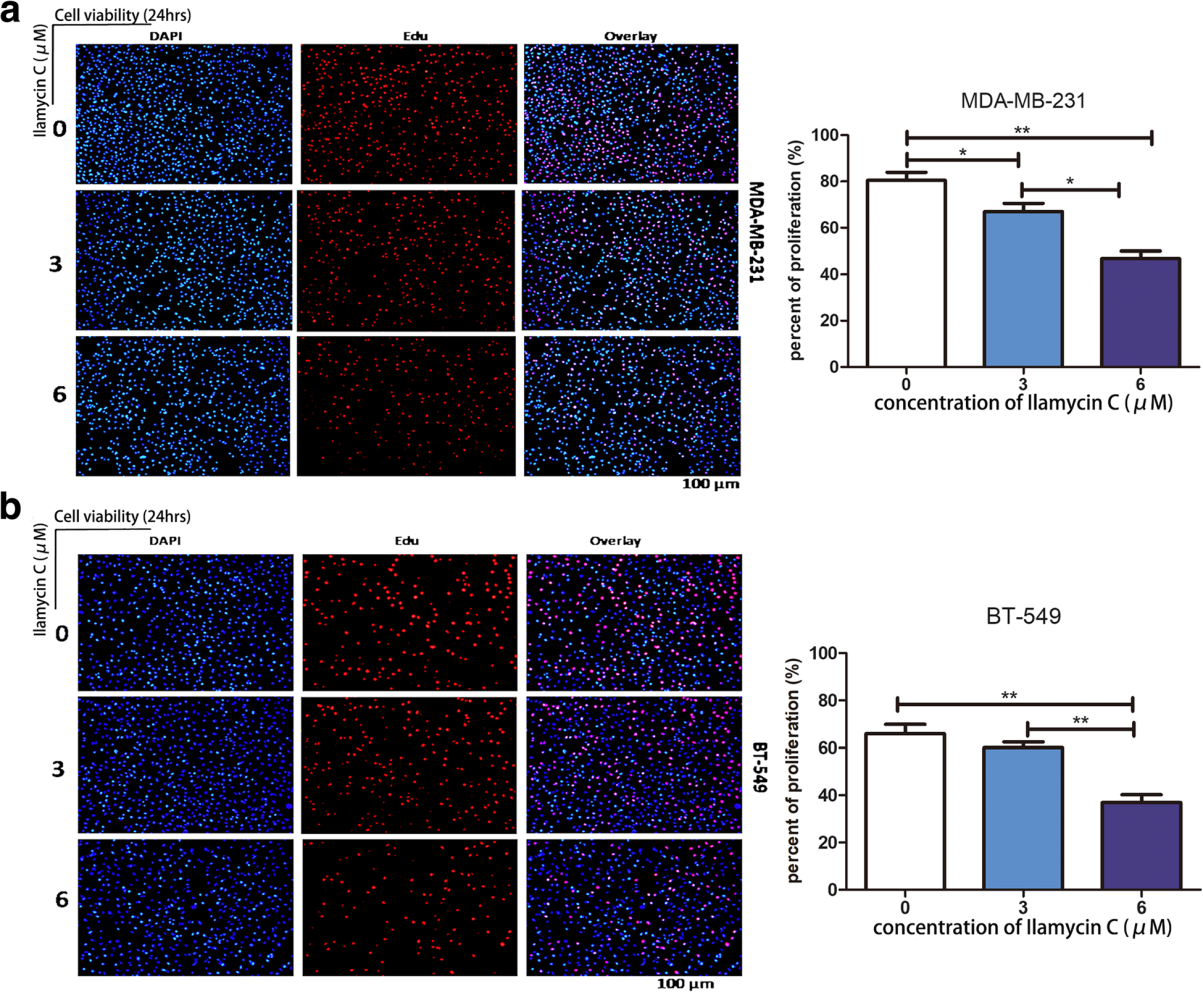
Ilamycin C suppresses proliferation in TNBC cells. **a** MDA-MB-231 and **b** BT-549 cells were treated for 24 h with vehicle control, 3 μM and 6 μM of Ilamycin C respectively and analyzed by EdU assay. Experiments were performed in triplicates. **p < 0.01, *p < 0.05

Since Ilamycin C decreased TNBC cell viability effectively, we next examined the suppressive effect of Ilamycin C on TNBC cell proliferation by EdU incorporation assay. MDA-MB-231 and BT-549 cells were treated with different concentrations of Ilamycin C for 24 h. The results showed that the EdU-positive cells of both cell lines were significantly decreased when treated with Ilamycin C at 6 μM, indicating Ilamycin C can suppress cell proliferation in TNBC (Fig. 2).

### Ilamycin C induces apoptosis in TNBC cells

**Fig. 3:**
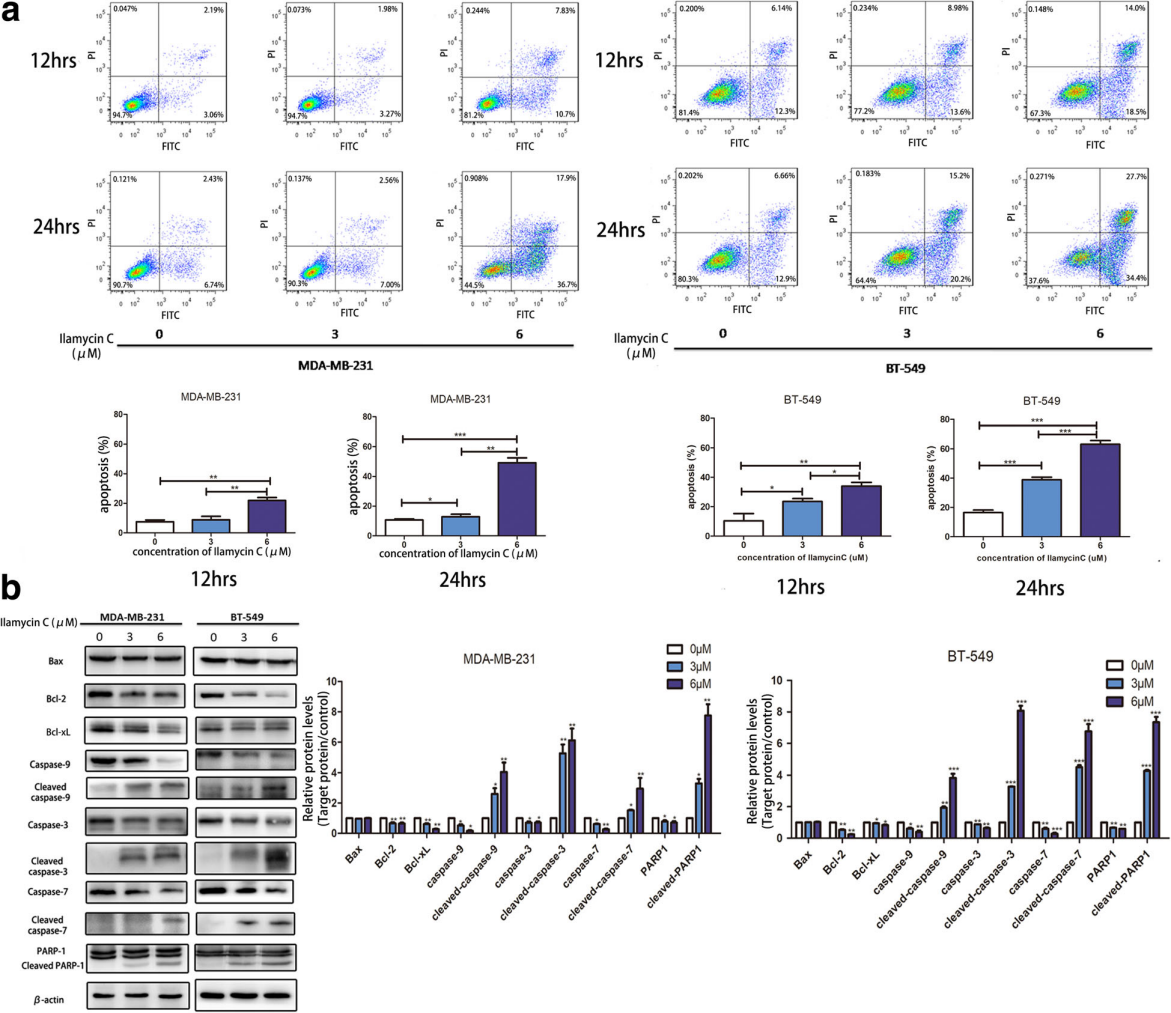
Ilamycin C induces apoptosis in TNBC cells. **a** The percentage of apoptotic cells were analyzed by flow cytometry. **b** The expression levels of apoptosis-related proteins were analyzed by western blot. Experiments were performed in triplicates. ***p < 0.001, **p < 0.01, *p < 0.05

The effect of Ilamycin C on TNBC apoptosis was further investigated and analyzed by flow cytometry after treating MDA-MB-231 and BT-549 cells with 0, 3, and 6 μM for 12 h and 24 h. Flow cytometry results demonstrated Ilamycin C induced cell apoptosis, and MDA-MB-231 cells showed a significant rise in apoptosis from 5.3% in vehicle control to 18.5% at 12 h and from 9.6% in vehicle control to 54.6% at 24 h when treated with 6 μM Ilamycin C. Similar results were gained in BT-549 cells (Fig. 3a).

These findings were confirmed by examining apoptosis-related proteins in MDA-MB-231 and BT-549 cell lines. Anti-apoptotic Bcl-2 families, such as Bcl-xL and Bcl-2, interact with the pore-forming protein Bax to prevent the induction of mitochondrial outer membrane permeabilization (MOMP) and subsequent apoptosis; thus, an increased ratio of Bax/Bcl-2 signifies the induction of apoptosis in cells [22]. Downregulated anti-apoptotic Bcl-2 protein family can activate caspase-9 and further activate caspase-3 and caspase-7 in an intrinsic apoptotic process [23]. PARP1 is an essential apoptotic protein and can be cleaved at the onset of apoptosis by caspase-3 or caspase-7 [24–27]. The western blot results showed that Ilamycin C increased the levels of cleaved caspase-3,7,9 and PARP1 proteins in both TNBC cell lines, whereas it reduced Bcl-2 and Bcl-xL (Fig. 3b). Although Bax was found unaltered in both cell lines when treated with Ilamycin C, the increased ratio of Bax/Bcl-2 and accumulation of cleaved caspase-3,7,9 and PARP1 validated the occurrence of apoptosis in TNBC cells. These results together with flow cytometry data indicated that Ilamycin C can induce apoptosis in TNBC cells partially via Bax/Bcl-2-related caspase-dependent apoptosis pathway.

### Ilamycin C inhibits migration and invasion in TNBC cells

**Fig. 4:**
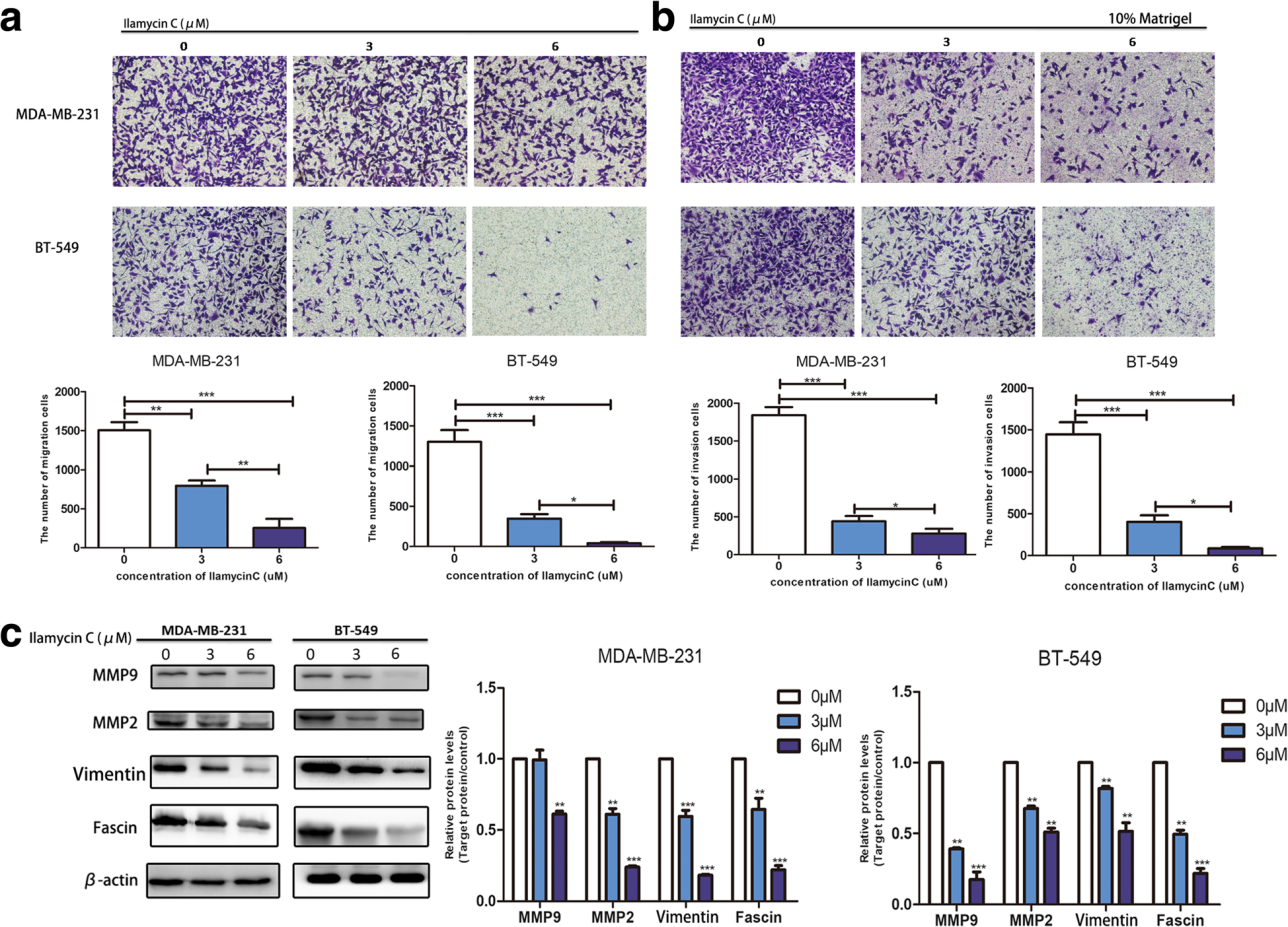
Ilamycin C inhibits migration and invasion in TNBC cells. After 24 h exposure of MDA-MB-231 and BT-549 cells to Ilamycin C, **a** migration assay, **b** invasion assay, and **c** western blot assay were performed. Experiments were performed in triplicates. ***p < 0.001, **p < 0.01

The fact that TNBC is the most aggressive subtype of breast cancer led us to further explore the effect of Ilamycin C on migration and invasion in TNBC cells. We utilized transwell migration and Matrigel invasion assays to assess the migratory and invading capacity of MDA-MB-231 and BT-549 cell lines following the treatment of Ilamycin C after 24 h. The migration assay (Fig. 4a) demonstrated that the migration abilities of both TNBC cells were significantly weakened in a dose-dependent pattern, especially after the treatment of ilamycin C at the concentration of 6 μM compared with the untreated group. For the invasion assay, the significantly reduced number of cells that invaded through Matrigel to the undersurface of transwell filter was observed in both TNBC cell lines when treated with gradually increasing concentration of Ilamycin C (Fig. 4b), demonstrating Ilamycin C could cause a dose-dependent reduction in invasion of both TNBC cells.

Matrix metalloproteinase (MMP) family such as MMP2 and MMP9 [28], the vital epithelial-mesenchymal transition (EMT)-related factor vimentin [29], and the actin-bundling protein fascin [30] play essential roles in breast cancer metastasis. Thus, these proteins were detected to further validate the inhibitory effect of Ilamycin C on migration and invasion in TNBC cells using western blot analysis in this study. Results showed that the expressions of MMP2, MMP9, vimentin, and fascin were significantly decreased in the presence of Ilamycin C treatment in a dose-dependent manner (Fig. 4c). These findings suggested that Ilamycin C could effectively inhibit invasion and migration through suppressing the levels of MMPs, vimentin, and fascin in TNBC.

### Ilamycin C suppresses the IL-6/STAT3 pathway in TNBC cells

**Fig. 5:**
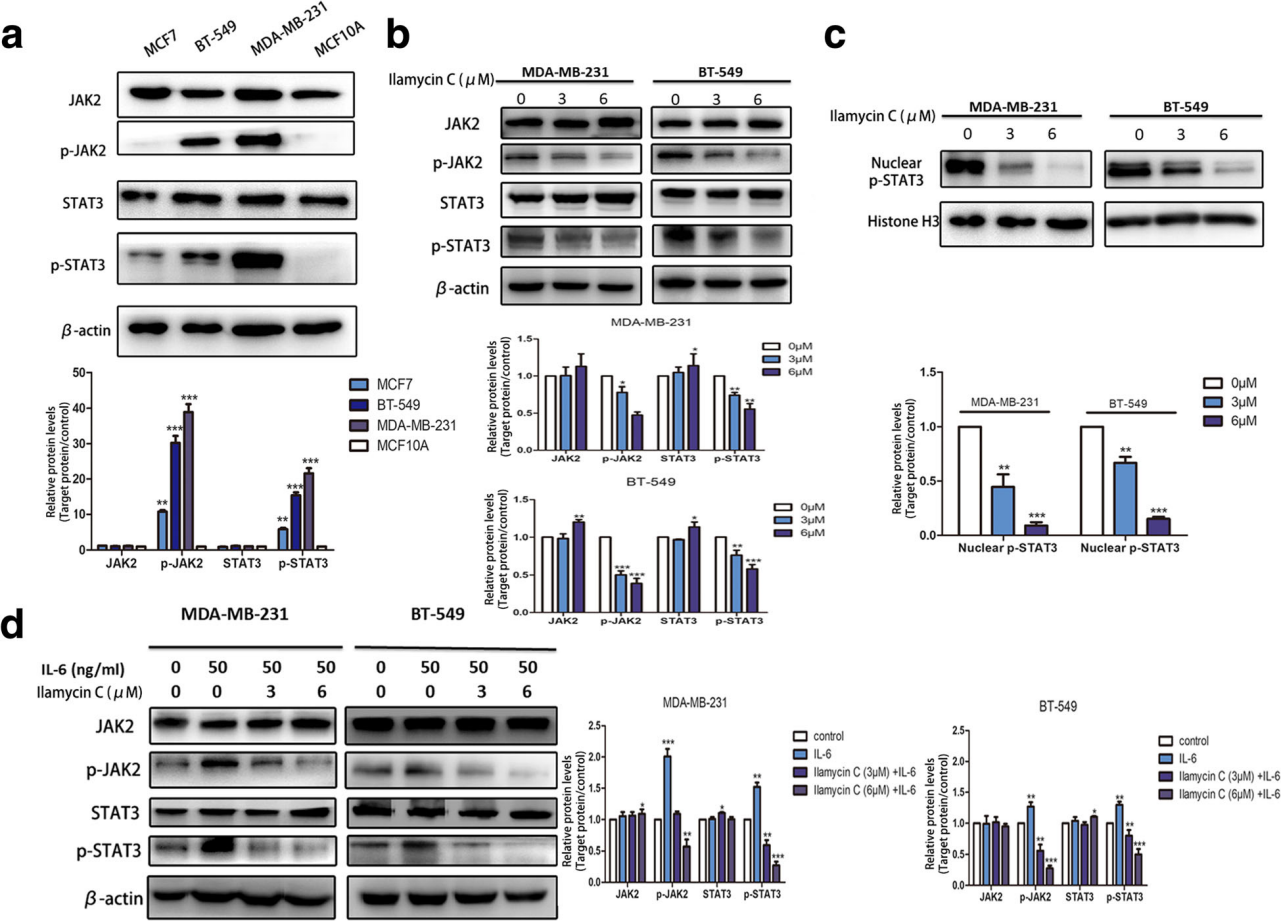
Ilamycin C suppresses IL-6/STAT3 pathway in TNBC cells. **a** The expression of basal p-JAK2 and p-STAT3 in TNBC cell lines (MDA-MB-231, BT-549), non-TNBC cell lines (MCF7), and normal breast cell lines (MCF10A). **b** The basal p-JAK2 and p-STAT3 were reduced after the treatment of Ilamycin C for 24 h. **c** Decreased level of p-STAT3 in nucleus after the treatment of Ilamycin C for 24 h. **d** Ilamycin C prevented IL-6-induced p-JAK2 and p-STAT3 in MDA-MB-231 and BT-549 cells after the treatment of Ilamycin C for 24 h

Recent studies showed that activated STAT proteins, especially STAT3, are involved in the progression of many malignant tumors [31]. The suppression of phosphorylated STAT3 (p-STAT3) can induce apoptosis and inhibit metastasis in cancer [32, 33]. In TNBC, poor prognosis and chemotherapy resistance are related to the activation of STAT3 [34]. It has been reported that activated STAT3 was mainly found in TNBC cells [13]. This is consistent with our results that basal p-STAT3 and its upstream protein p-JAK2 were significantly higher in TNBC cells (MDA-MB-231 and BT-549) than non-TNBC cells (MCF7), and undetectable in normal breast cells (MCF10A) (Fig. 5a). We further confirmed that after the exposure to Ilamycin C for 24 h, the levels of basal p-JAK2 and p-STAT3 were decreased in a dose-dependent pattern, whereas the total JAK2 and STAT3 was unaltered in both TNBC cell lines (Fig. 5b).

Studies also demonstrated that only the p-STAT3, rather than STAT3, can translocate to the cell nucleus and play a regulatory role by directly binding to the specific promotor region of targets [35]. To investigate whether Ilamycin C could block the function of p-STAT3 through decreasing the level of p-STAT3 in the nucleus, the level of p-STAT3 in the cell nucleus was examined using extracted nuclear proteins of MDA-MB-231 and BT-549 treated with or without Ilamycin C for 24 h, and nuclear Histone H3 was used as control. Results showed that Ilamycin C led to a significant decrease of the p-STAT3 level in the nucleus (Fig. 5c), revealing Ilamycin C could suppress the function of p-STAT3 through decreasing the level of p-STAT3 in the nucleus in TNBC cells.

It has been proved that JAK2/STAT3 can be activated by many upstream proteins including IL-6 [8–10], and the inhibition of IL-6/JAK2/STAT3 signaling activation can suppress the aggressiveness of TNBC [36, 37]. To explore the underlying mechanism of Ilamycin C, we investigated whether Ilamycin C can inhibit IL-6-induced activation of JAK2/STAT3 in TNBC. Cells of MDA-MB-231 and BT-549 were pretreated with Ilamycin C in different concentrations for 24 h before exposing to 50 ng/mL IL-6 for 30 min. As shown in Fig. 5d, IL-6 induced p-JAK2 and p-STAT3 in both TNBC cell lines; however, Ilamycin C can prevent the increase of phosphorylation of the JAK2/STAT3 level. These results revealed that Ilamycin C may function as a novel inhibitor of the IL-6/JAK2/STAT3 signaling pathway.

### STAT3 overexpression rescues Ilamycin C-mediated effects of apoptosis, migration, and invasion in TNBC

**Fig. 6:**
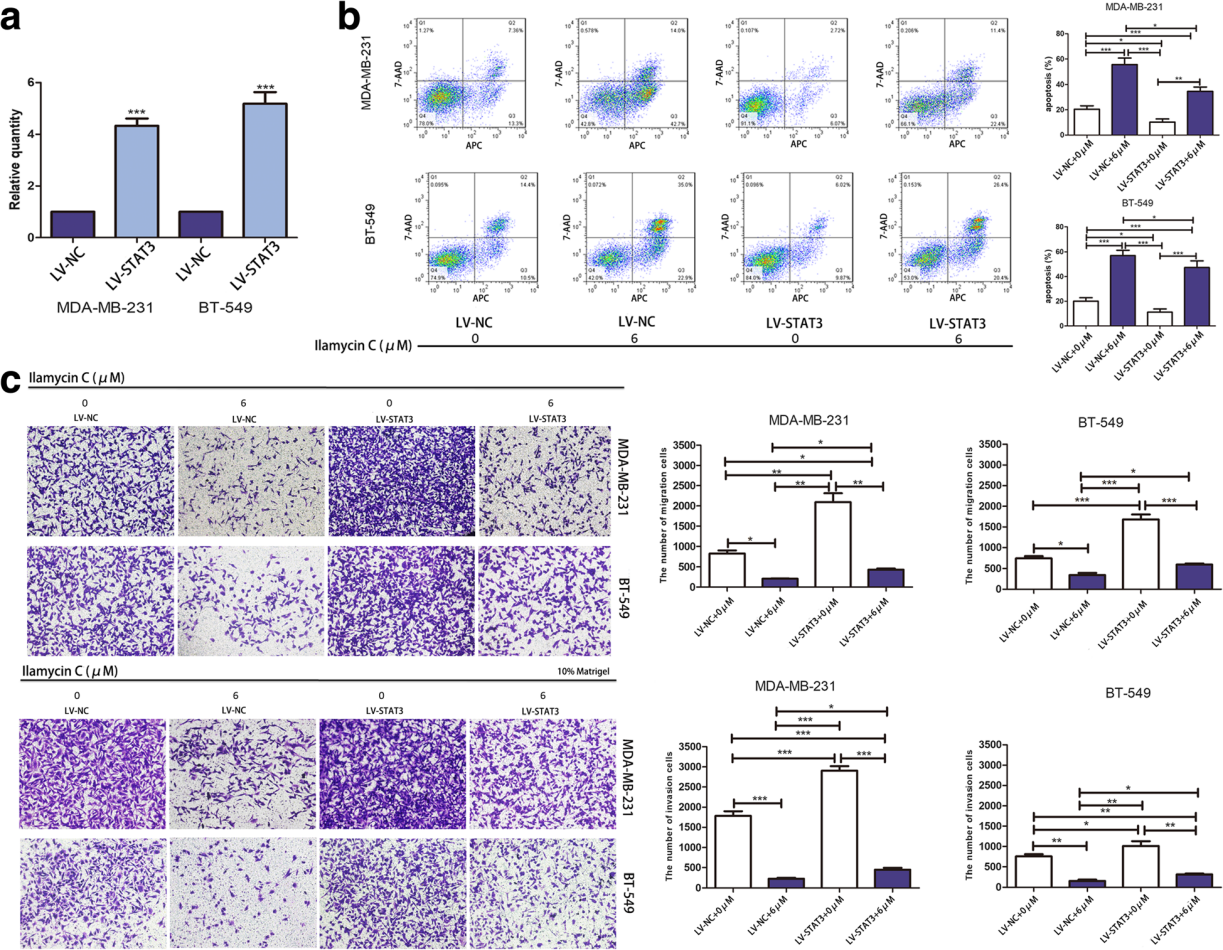
STAT3 overexpression rescues Ilamycin C-mediated effects of apoptosis, migration, and invasion in TNBC. **a** The relative mRNA expression of STAT3 in MDA-MB-231 and BT-549 after the infection with LV-STAT3 or LV-NC by qRT-PCR. **b** Overexpression of STAT3 reversed Ilamycin C-mediated apoptosis in TNBC cell lines. **c** Overexpression of STAT3 reversed Ilamycin C-mediated migration and invasion in TNBC cell lines

**Fig. 7:**
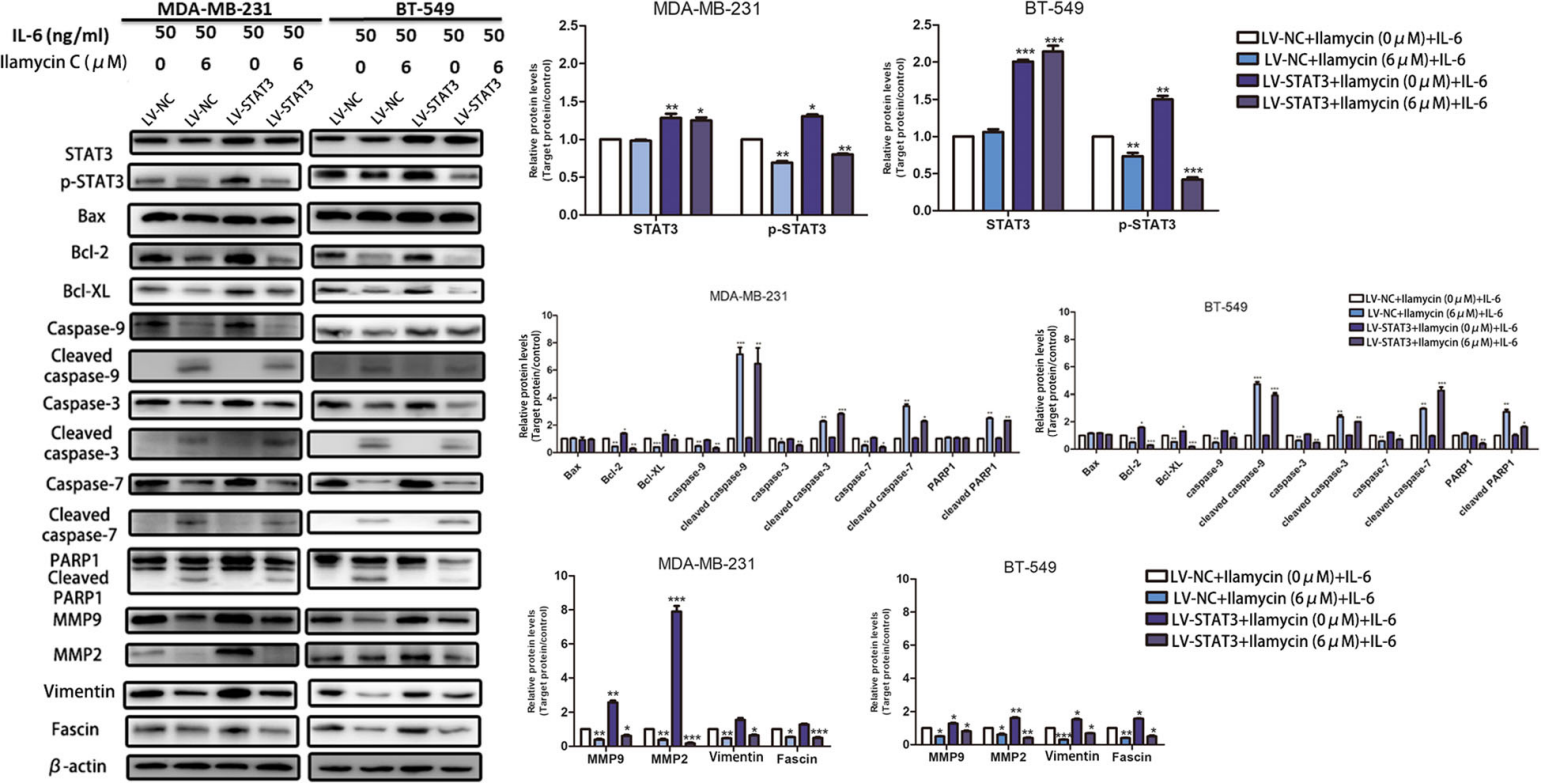
STAT3 overexpression rescues Ilamycin C-mediated effects in TNBC. The expressions of proteins of the IL-6-induced p-STAT3 pathway confirmed by western blot after the infection with lentivirus-STAT3 (LV-STAT3) or lentivirus-negative control (LV-NC) in MDA-MB-231 and BT-549 cells with or without Ilamycin C treatment

To investigate whether STAT3 is involved in Ilamycin C-mediated apoptosis, migration, and invasion in TNBC, a lentivirus system was utilized to stably overexpress STAT3. Increased STAT3 and p-STAT3 levels were confirmed by qRT-PCR after infecting with lentivirus-STAT3 (LV-STAT3) or lentivirus-negative control (LV-NC) in MDA-MB-231 and BT-549 cell lines (Fig. 6a). Our results showed that Ilamycin C could sharply promote apoptosis; however, under the same concentration of Ilamycin C, the apoptosis rates of cells overexpressing STAT3 were significantly decreased compared with that of cells infected with LV-NC (Fig. 6b). Similarly, Ilamycin C could suppress migration and invasion by a large margin, but the number of migration and invasion cells was significantly increased in cells overexpressing STAT3 compared with that of cells infected with LV-NC when treated with same concentration of Ilamycin C (Fig. 6c). These results demonstrated that overexpression of STAT3 reversed Ilamycin C-mediated apoptosis, migration, and invasion in TNBC cell lines. The expressions of proteins involved in apoptosis, migration, and invasion, which are downstream targets of p-STAT3, were also confirmed by western blot after infecting with lentivirus-STAT3 (LV-STAT3) or lentivirus-negative control (LV-NC) in MDA-MB-231 and BT-549 cells with or without Ilamycin C treatment (Fig. 7). These findings indicated that Ilamycin C exerted its important effects in TNBC through the inhibition of STAT3.

### Knockdown of STAT3 enhances Ilamycin C-mediated effects of apoptosis, migration, and invasion in TNBC

**Fig. 8:**
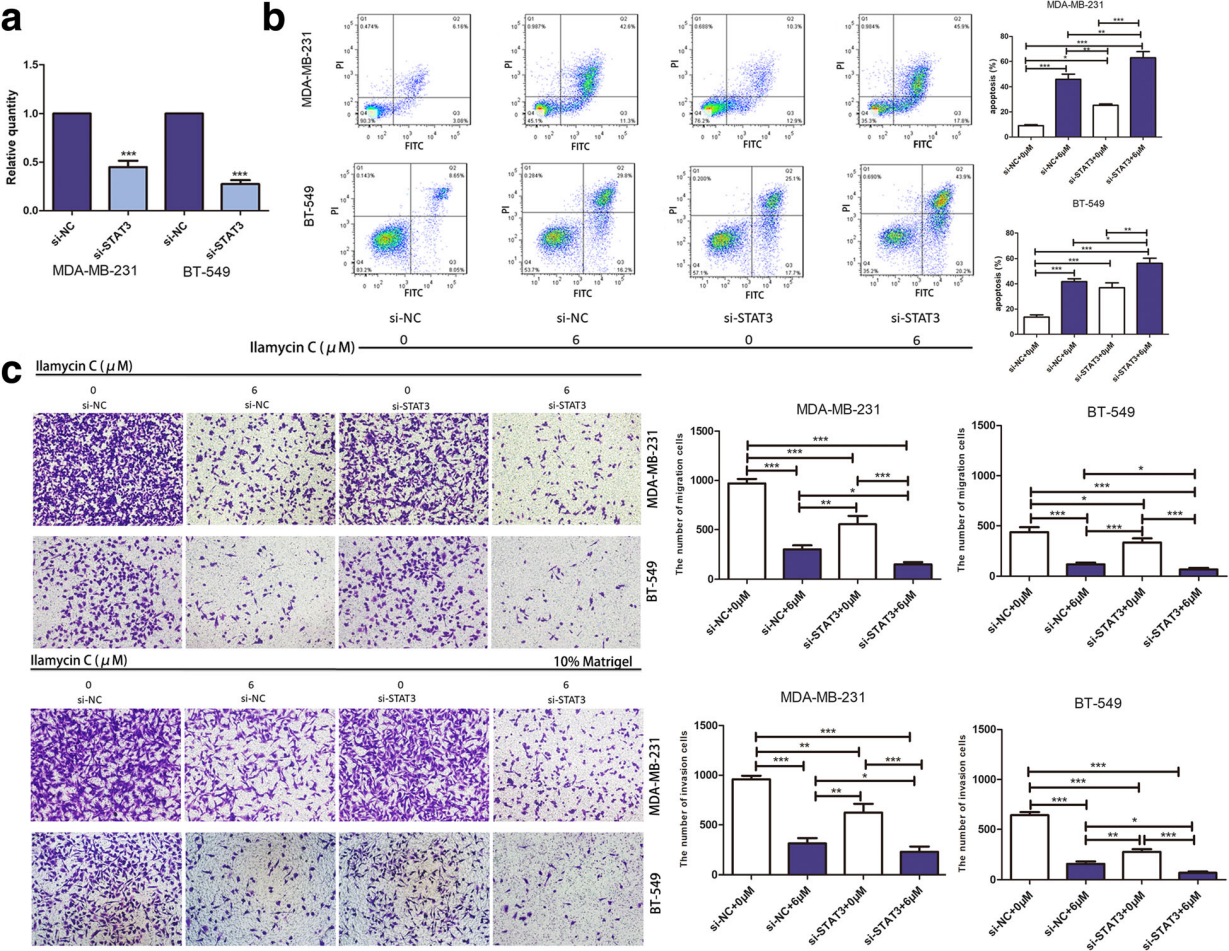
Knockdown of STAT3 enhances Ilamycin C-mediated effects of apoptosis, migration, and invasion in TNBC. **a** The relative mRNA expression of STAT3 in MDA-MB-231 and BT-549 after the transfection with si-STAT3 or si-NC by qRT-PCR. **b** Overexpression of STAT3-enhanced Ilamycin C-mediated apoptosis in TNBC cell lines. **c** Overexpression of STAT3 enhanced Ilamycin C-mediated migration and invasion in TNBC cell lines

**Fig. 9:**
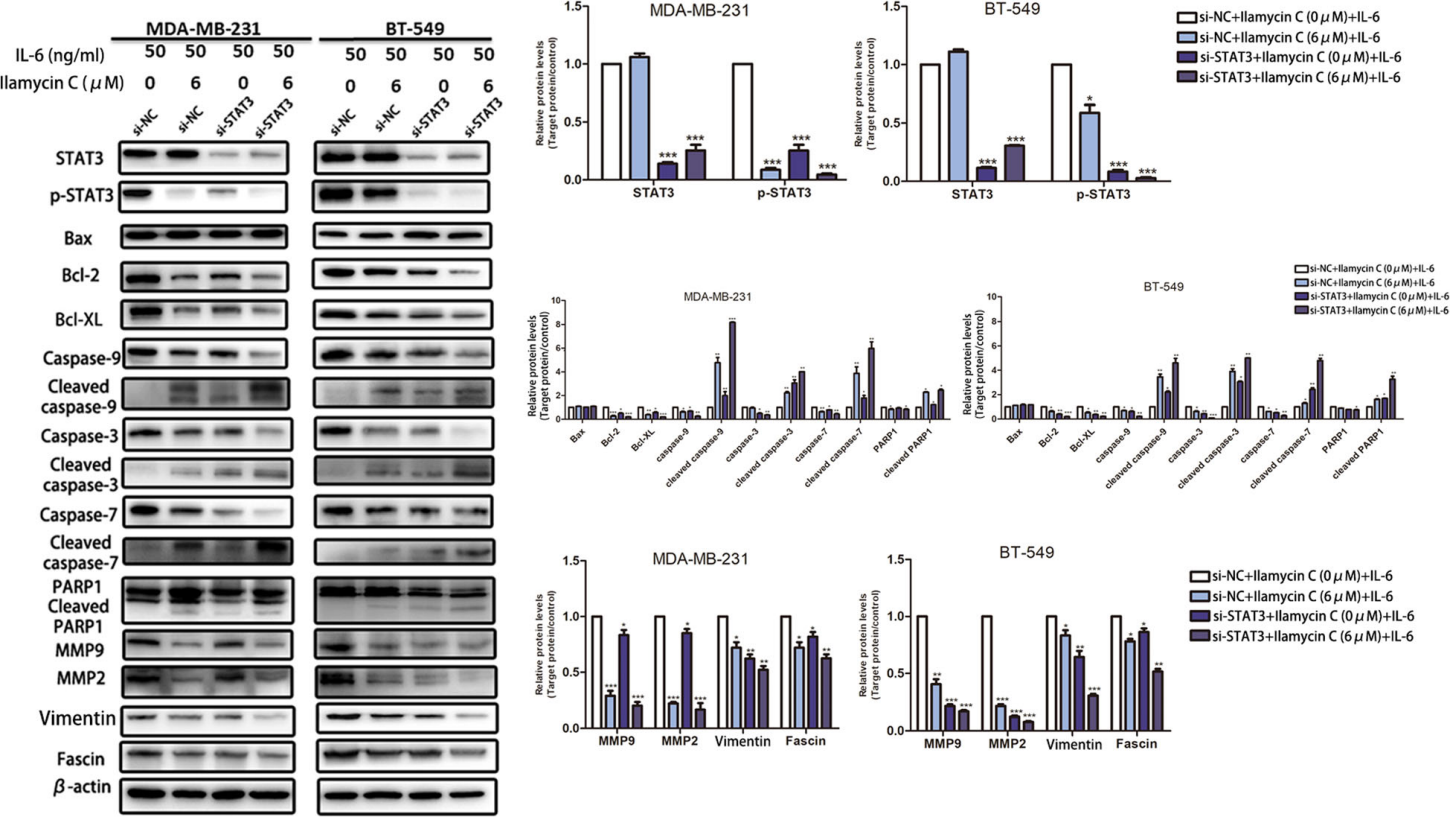
Knockdown of STAT3 enhances Ilamycin C-mediated effects in TNBC. The expressions of proteins of the IL-6-induced p-STAT3 pathway confirmed by western blot after the transfection with si-STAT3 or si-NC in MDA-MB-231 and BT-549 cells with or without Ilamycin C treatment

To further determine whether knockdown of STAT3 can enhance Ilamycin C-mediated effects, we transfected TNBC cells with STAT3 RNA interference (RNAi) to knock down STAT3. The knockdown efficiency of STAT3 was examined in MDA-MB-231 and BT-549 (Fig. 8a). As expected, results showed that knockdown of STAT3 significantly enhanced Ilamycin C-mediated effects of apoptosis, migration, and invasion in TNBC cells (Fig. 8b, c). We also confirmed the expressions of the IL-6/STST3 pathway-related proteins involved in apoptosis, migration, and invasion by western blot after transfecting with si-STAT3 or si-negative control (si-NC) in MDA-MB-231 and BT-549 cells with or without Ilamycin C treatment (Fig. 9). These results together with that of STAT3 overexpression provided the evidence that Ilamycin C could induce apoptosis and inhibit migration and invasion by regulating the IL-6/STAT3 pathway in TNBC.

## Discussion

TNBC is associated with higher metastasis and poorer prognosis compared with other breast cancer subtypes due to the lack of effective chemotherapeutic drugs and frequently acquired chemoresistance [38, 39]. Hence, the development of novel drugs that can selectively and specifically target TNBC cells is urgently needed. In recent years, marine-derived natural products have been found to have better antitumor activities against many kinds of cancer, thus providing a constant source for new drug discovery against cancer [40–42]. Our previous study showed that Ilamycin C, a novel compound separated from the deep South China Sea-derived *S. atratus* SCSIO ZH16, has a strong cytotoxic activity against several cancers including breast cancer cell line MCF7 [21]. However, the cytotoxic activity of Ilamycin C in TNBC cells has not yet been tested, and the detailed antitumor mechanism remains unknown. In this work, we tested the cytotoxic activity of Ilamycin C in TNBC cell lines (MDA-MB-231 and BT-549), non-TNBC cell line (MCF7), and normal breast epithelial cell line (MCF10A). Doxorubicin and cisplatin are the traditional clinical chemotherapy drugs for TNBC [43]. Interestingly, compared with doxorubicin and cisplatin, the IC_50_ values revealed that the cytotoxic activity of Ilamycin C is more specific to TNBC cells than non-TNBC MCF7 and nonmalignant MCF10A cells, implying Ilamycin C may play a selective inhibitory role in TNBC, implying Ilamycin C has potential to serve as a novel clinical chemotherapy drug for the treatment of TNBC.

Bcl-2 family members consist of pro-apoptotic and anti-apoptotic proteins, which are crucial to control apoptosis. Besides Bcl-2, the Bcl-xL, another member of the Bcl-2 family, is known as anti-apoptosis proteins involved in the suppression of caspase activation [23]. Studies also showed that the caspase family is involved in extrinsic and intrinsic apoptotic pathways and caspase-9, caspase-3, and caspase-7 can be sequentially activated by the Bcl-2 protein family, further cleaving the vital apoptotic protein PARP1 to trigger apoptosis [24–27]. Our results demonstrated that Ilamycin C could significantly promote the apoptosis of TNBC cells at 6 μM after the treatment for 12 h and 24 h by the decreased interaction of the Bcl-2 family with Bax due to decrease of Bcl-2 and Bcl-xL and consequent activation of caspase-3,7,9 and PARP1.

In TNBC patients, poor prognosis is related to the characteristics of strong invasion and migration ability of TNBC cells [44]. We observed that the migration and invasion of MDA-MB-231 and BT-549 cells were suppressed even in the presence of Ilamycin C at 3 μM for 24 h. It has been reported that MMPs are major components involved in metastasis, especially, the increased MMP2 and MMP9, two important members of MMPs, are associated with cancer aggressiveness and metastasis in TNBC [45, 46]. Studies also showed that EMT is an initial step in cancer metastasis, and the major cytoskeletal protein vimentin, which is a positive regulator and a canonical marker of EMT, is correlated with aggressive clinical phenotype in TNBC [47]. Fascin is an actin-bundling protein of cytoskeleton, and upregulated fascin can promote migration and invasion in cancer metastasis including TNBC [30]. Our results found that Ilamycin C reduced the expressions of MMP2, MMP9, vimentin, and fascin in both MDA-MB-231 and BT-549 cell lines, suggesting that Ilamycin C could inhibit cell invasion and migration through the suppression of MMPs, vimentin, and fascin in TNBC.

Increasing studies showed that STAT3 is an essential gene that participates in cancer cell proliferation, apoptosis, metastasis, and other cellular events including EMT [48–52]. Notably, p-STAT3, activated by JAK2, was found in approximately 80% of TNBC cells, indicating that STAT3 could be an attractive novel therapeutic target for TNBC [13]. Activated STAT3 dimerizes and translocates to cell nucleus and directly binds to the specific promotor region of targets, such as Bcl-2 families, MMP2, MMP9, vimentin, and fascin leading to transcriptional activation of them [30, 47, 52–54]. Consistent with the reported finding that activated STAT3 was mainly found in TNBC cells [13], our results found that basal p-STAT3 and its upstream protein p-JAK2 were high in TNBC cells (MDA-MB-231 and BT-549), while weak in non-TNBC cells (MCF7) and undetectable in normal breast cells (MCF10A). Moreover, we confirmed Ilamycin C could block the function of p-STAT3 through decreasing the level of p-STAT3 in the nucleus. STAT3 can be phosphorylated by activated JAK2 induced by IL-6, which is a key mediator of the inflammatory response and functions as a crucial regulator in the progression of breast cancer [55, 56]. In this study, treatment with Ilamycin C at 6 μM significantly reduced the levels of phosphorylated JAK2 and STAT3 and their downstream proteins involved in apoptosis, migration, and invasion in both MDA-MB-231 and BT-549 cell lines, suggesting that Ilamycin C can function as an effective inhibitor of the IL-6/STAT3 pathway in TNBC cells. Notably, we further validated that STAT3 overexpression could rescue and knockdown of STAT3 could enhance Ilamycin C-mediated effects of apoptosis, migration, and invasion in TNBC. Taken together, these findings provided the evidence that Ilamycin C could induce apoptosis and inhibit migration and invasion by suppressing the IL-6/STAT3 pathway in TNBC.

**Fig. 10:**
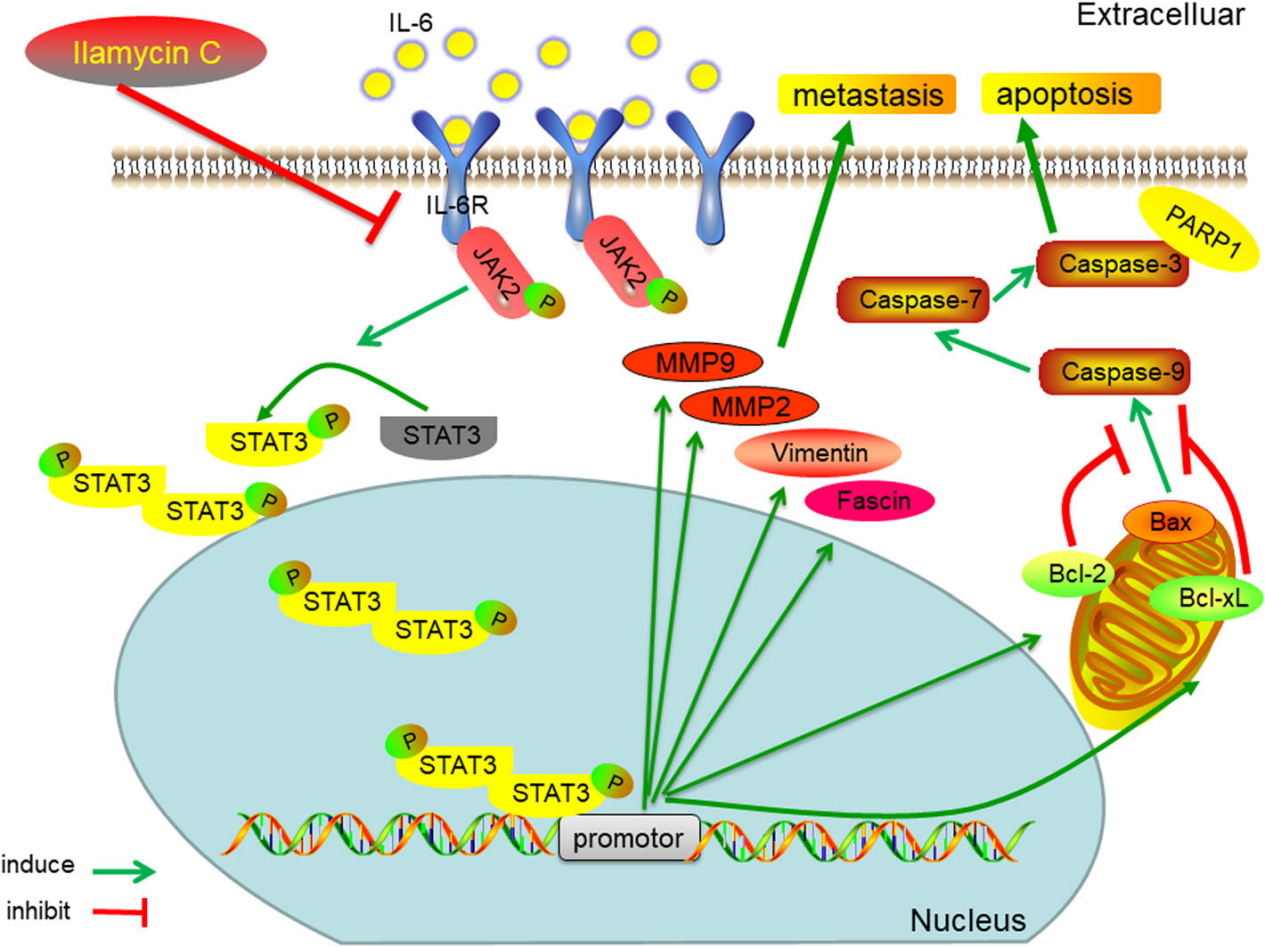
Schematic presentation of the anti-TNBC mechanism of Ilamycin C. The inhibition of IL-6 induced JAK2/STAT3 phosphorylation by Ilamycin C abrogates the function of p-STAT3 through decreasing the level of p-STAT3 in the nucleus, thus further regulating the expressions of its downstream target genes, which ultimately contributed to promote Bax/Bcl-2-related caspase-dependent apoptosis and suppress migration and invasion through MMP2/MMP9/vimentin/fascin in TNBC cells

Based on our and reported findings, we propose that the inhibition of IL-6-induced JAK2/STAT3 phosphorylation by Ilamycin C abrogates the function of p-STAT3 through decreasing the level of p-STAT3 in the nucleus, thus regulating the expressions of its downstream target genes, which ultimately contributed to promote Bax/Bcl-2-related caspase-dependent apoptosis and suppress migration and invasion through MMP2/MMP9/vimentin/fascin in TNBC cells (Fig. 10). For the aim of developing a novel promising drug candidate for the treatment of TNBC, the in vivo activity of Ilamycin C will be further studied.

## Conclusions

This study found that Ilamycin C has more preferential cytotoxicity in TNBC cells than non-TNBC MCF7 and nonmalignant MCF10A cells. Further investigation revealed the mechanism that Ilamycin C can induce apoptosis and inhibit invasion and migration in TNBC by suppressing IL-6-induced STAT3 phosphorylation. This study provides the first evidence that Ilamycin C has the potential as a novel IL-6/STAT3 inhibitor for TNBC treatment in the future.

## Additional file

Additional file 1: **Figure S1.** Structure of ilamycin C. (TIF 49 kb)

## Data Availability

All authors ensure that all data generated or analyzed during this study are included in this published article.

## Abbreviations

TNBC: Triple-negative breast cancer
ER: Estrogen receptor
PR: Progesterone receptor
HER2: Human epidermal growth factor receptor 2
STAT3: Signal transducer and activator of transcription-3
MOMP: Mitochondrial outer membrane permeabilization
MMP: Matrix metalloproteinase
EMT: Epithelial-mesenchymal transition
